# Dynamic QTL mapping revealed primarily the genetic structure of photosynthetic traits in castor (*Ricinus communis* L.)

**DOI:** 10.1038/s41598-023-41241-y

**Published:** 2023-08-28

**Authors:** Guanrong Huang, Xuegui Yin, Jiannong Lu, Liuqin Zhang, Dantong Lin, Yu Xie, Haiyan Liu, Chaoyu Liu, Jinying Zuo, Xiaoxiao Zhang

**Affiliations:** College of Coastal Agricultural Sciences, Guangdong Ocean University, Zhanjiang, 524088 China

**Keywords:** Agricultural genetics, Genetic linkage study, Genetic markers, Heritable quantitative trait, Plant genetics

## Abstract

High photosynthetic efficiency is the basis of high biomass and high harvest index in castor (*Ricinus communis* L.). Understanding the genetic law of photosynthetic traits will facilitate the breeding for high photosynthetic efficiency. In this study, the dynamic QTL mapping was performed with the populations F_2_ and BC_1_ derived from 2 parents with significant difference in net photosynthetic rate (Pn) at 3 stages, in order to reveal the genetic structure of photosynthetic traits. In F_2_ population, 26 single-locus QTLs were identified, including 3/3/1 (the QTL number at stage I/II/III, the same below), 1/2/0, 1/2/2, 1/3/1, 0/1/1, and 1/1/2 QTLs conferring Pn, water use efficiency (Wue), transpiration rate (Tr), stomatal conductance (Gs), intercellular CO_2_ concentration (Ci) and chlorophyll content (Cc), with a phenotypic variation explained (PVE) of 8.40%/8.91%/6.17%, 5.36%/31.74%/0, 7.31%/12.80%/15.15%, 1.60%/6.44%/0.02%, 0/1.10%/0.70% and 2.77%/3.96%/6.50% respectively. And 53 epistatic QTLs (31 pairs) were identified, including 2/2/5, 5/6/3, 4/4/2, 6/3/2, 3/2/0 and 4/0/0 ones conferring the above 6 traits, with a PVE of 6.52%/6.47%/19.04%, 16.72%/15.67%/14.12%, 18.57%/15.58%/7.34%, 21.72%/8.52%/7.13%, 13.33%/4.94%/0 and 7.84%/0/0 respectively. The QTL mapping results in BC_1_ population were consistent with those in F_2_ population, except fewer QTLs detected. Most QTLs identified were minor-effect ones, only a few were main-effect ones (PVE > 10%), focused on 2 traits, Wue and Tr, such as *qWue1.1*, *qWue1.2*, *FqTr1.1, FqTr6, BqWue1.1* and *BqTr3*; The epistatic effects, especially those related to the dominance effects were the main genetic component of photosynthetic traits, and all the epistatic QTLs had no single-locus effects except *qPn1.2*, *FqGs1.2, FqCi1.2* and *qCc3.2*; The detected QTLs underlying each trait varied at different stages except stable QTLs *qGs1.1*, detected at 3 stages, *qWue2*, *qTr1.2* and *qCc3.2*, detected at 2 stages; 6 co-located QTLs were identified, each of which conferring 2–5 different traits, demonstrated the gene pleiotropy between photosynthetic traits; 2 QTL clusters, located within the marker intervals RCM1842-RCM1335 and RCM523-RCM83, contained 15/5 (F_2_/BC_1_) and 4/4 (F_2_/BC_1_) QTLs conferring multiple traits, including co-located QTLs and main-effect QTLs. The above results provided new insights into the genetic structure of photosynthetic traits and important references for the high photosynthetic efficiency breeding in castor plant.

## Introduction

Castor plant is an important industrial oil crop, with a seed oil content of 46–55%^1^. Castor oil is widely used in the medicinal, biodiesel, and specialty chemical fields due to its high amount of ricinoleic acid, which has exceptionally distinctive physical and chemical properties^2^. Castor oil has been in short supply as a result of the economy growth since its production is well below the level of global demand^3^. Castor cultivation is the only commercial source of castor oil, but with poor yields and high labor costs, the cultivation area is decreasing year by year. Thus, it is an urgent need to cultivate high-yielding varieties to promote the growth of castor industry at the current stage.

Enhancing crop photosynthesis can significantly boost yield and biomass^4, 5^. However, fewer researches have been reported for photosynthetic traits than other traits like yield and plant type, mostly due to the difficulty of data collecting and the environmental effects of the measurement process^6^. The improvement of instruments and measurement methods has promoted the study of photosynthetic traits, especially in model plants, a number of genes controlling photosynthetic traits have been found, for example, the genes mediating water use efficiency (Wue)^7–11^, transpiration rate (Tr)^12–14^ and stomatal density^15, 16^ in *Arabidopsis*, and the genes regulating photosynthetic capacity and Wue in rice^17–20^. In addition, some QTLs underlying photosynthetic traits have been successfully detected in other crops, but with low contribution rate (most of them with contribution rate less than 10%)^21–24^. Such a situation might be caused by the epistatic effect partially masking the single-locus effect and the joint control of photosynthetic traits by many minor-effect genes^22^. Nevertheless, the additional insertion of *OsDREB1C* resulted in the enhancement of photosynthetic capacity, which increased yield by more than 40% in rice^18^. It strongly demonstrated the huge potential of regulating the expression of major genes controlling photosynthetic traits by genetic engineering to raise crop yields.

A little was reported on photosynthetic traits in castor. Previous studies have reported that photosynthetic O_2_-evolution and ^14^CO_2_-fixation rates in blades decreased with increasing nuclear genome duplication^25^; Photosynthesis in capsule wall promotes fuller seed filling^26^, and that in seed coat increases lipid accumulation^27^, seedling growth^28^ and seed development^29^; In response to stresses such as high temperature, drought, flooding and high salinity, castor plants reduce water loss by decreasing stomatal conductance to prevent irreversible leaf wilting and further develop mechanisms to adapt to the environment^30–34^; *RcDREB1*, a pollen-specific and desiccation-associated transcription factor gene from castor, enhanced the drought tolerance (lower Tr), photosynthetic rate, biomass and pollen grain viability of transgenic tobacco^35^. The information concerning the genetic mechanism of photosynthetic traits is a serious shortage in castor. It is now necessary to identify the natural variation of photosynthetic traits in different populations and harness it through marker-assisted breeding techniques.

In this study, the dynamic mapping of QTLs conferring photosynthetic traits was conducted using populations F_2_ and BC_1_ to reveal the genetic structure of photosynthetic traits and provide guide for high photosynthetic efficiency breeding in castor.

## Results

### Descriptive statistics

**Figure 1:**
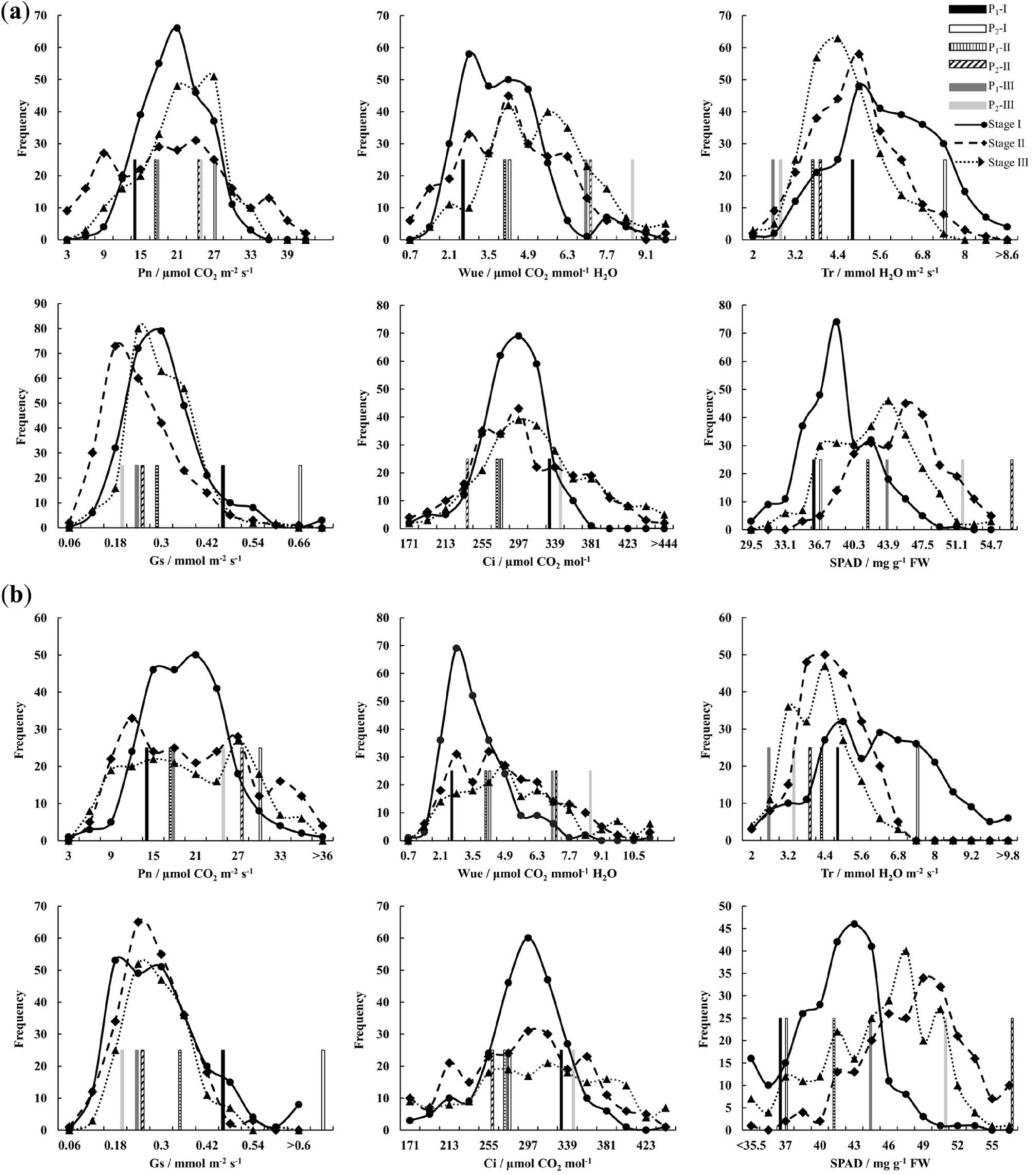
Frequency distribution of photosynthetic traits in populations F_2_ (**a**) and BC_1_ (**b**). The trait description is the same as in Table 1.

The Pn and Wue of P_2_ was significantly higher than P_1_ at 3 stages (*p* < 0.05) (Table 1). Cc was higher at 3 stages but significant only at stage II. The relative level of Tr, Gs and Ci between parents varied at different stages. In segregating populations, all the 6 traits behaved transgressive inheritance on both sides, and a multi-peaked or a skewed continuous distribution at 3 stages, demonstrated their characteristics of quantitative traits and suggested the existence of major genes (Fig. 1)^36^.

**Table 1:** Descriptive statistics of photosynthetic traits.

Trait^a^	Stage	Parents			F_2_ population					BC_1_ population				
		9048	16–201	Difference	Range	Mean ± SD	CV (%)	Skewness	Kurtosis	Range	Mean ± SD	CV (%)	Skewness	Kurtosis
Pn	I	14.39	28.36	− 13.97**	5.80–32.27	18.92 ± 5.07	26.82	− 0.02	− 0.34	2.79–36.31	18.01 ± 5.58	30.97	0.27	0.42
	II	17.34	25.44	− 8.10**	1.03–43.10	18.38 ± 9.40	51.14	0.18	− 0.66	4.33–39.14	19.27 ± 8.58	44.50	0.27	− 0.94
	III	16.13	23.30	− 7.17**	3.72–34.32	20.18 ± 6.10	30.22	− 0.37	− 0.20	3.07–35.13	18.47 ± 8.01	43.35	0.07	− 1.03
Wue	I	2.83	4.07	− 1.24**	0.89–8.77	3.68 ± 1.44	39.23	0.96	1.28	0.65–11.46	3.31 ± 1.41	42.54	1.55	4.60
	II	4.09	7.05	− 2.96*	0.21–9.57	3.97 ± 1.84	46.26	0.26	− 0.24	1.07–13.70	4.64 ± 2.22	47.82	0.90	1.47
	III	7.13	8.45	− 1.32*	0.72–9.78	4.95 ± 1.74	35.20	0.17	− 0.09	0.86–27.59	5.05 ± 2.92	57.86	2.90	18.74
Tr	I	5.18	7.04	− 1.86*	2.00–10.04	5.52 ± 1.43	25.96	0.12	− 0.27	1.90–11.80	5.91 ± 1.90	32.12	0.17	− 0.24
	II	4.27	3.82	0.45	1.84–8.38	4.58 ± 1.21	26.45	0.31	0.00	0.84–6.78	4.31 ± 1.00	23.26	− 0.20	0.39
	III	2.29	2.75	− 0.47**	1.95–7.16	4.25 ± 0.99	23.35	0.43	0.08	1.00–6.60	3.89 ± 0.99	25.41	0.06	− 0.02
Gs	I	0.49	0.63	− 0.14	0.10–0.77	0.28 ± 0.10	35.76	1.36	3.65	0.08–1.09	0.28 ± 0.14	48.67	1.97	6.75
	II	0.32	0.25	0.07*	0.05–0.66	0.23 ± 0.10	43.68	0.99	1.42	0.05–0.54	0.25 ± 0.09	33.69	0.42	0.30
	III	0.22	0.17	0.05**	0.08–0.57	0.28 ± 0.08	29.49	0.49	0.61	0.11–0.59	0.27 ± 0.08	30.61	0.64	0.61
Ci	I	331.75	265.71	66.04**	149.23–366.43	280.61 ± 35.19	12.54	− 0.53	1.00	119.53–459.45	283.05 ± 44.03	15.56	− 0.37	1.97
	II	269.62	246.64	22.98	150.43–462.43	294.47 ± 60.93	20.69	0.29	− 0.28	54.03–427.87	280.52 ± 65.21	23.25	− 0.38	0.32
	III	263.54	330.63	− 67.09**	106.47–511.60	307.20 ± 63.53	20.68	0.40	0.48	12.97–440.00	296.88 ± 74.10	24.96	− 0.45	0.34
Cc	I	36.22	36.64	− 0.42	28.20–50.70	37.81 ± 3.72	9.83	0.28	0.29	25.40–52.90	40.57 ± 3.99	9.84	− 0.65	1.50
	II	41.24	57.80	− 16.56**	33.50–54.60	44.44 ± 4.29	9.66	− 0.01	− 0.42	29.40–60.60	47.51 ± 4.45	9.36	− 0.33	0.70
	III	43.80	50.44	− 6.64	30.80–53.60	41.44 ± 4.42	10.68	0.11	− 0.22	25.50–56.90	44.27 ± 5.00	11.30	− 0.70	0.75

^a^*Pn* net photosynthetic rate/µmol CO_2_ m^−2^ s^−1^, *Wue* water use efficiency/μmol CO_2_ mmol^−1^ H_2_O, *Tr* transpiration rate/mmol H_2_O m^−2^ s^−1^, *Gs* stomatal conductance/mmol m^−2^ s^−1^, *Ci* intercellular CO_2_ concentration/µmol CO_2_ mol^−1^, *Cc* chlorophyll content/mg g^−1^ FW.

*^,^**Indicate the significant level at 0.05 and 0.01 respectively.

### Path analysis

**Figure 2:**
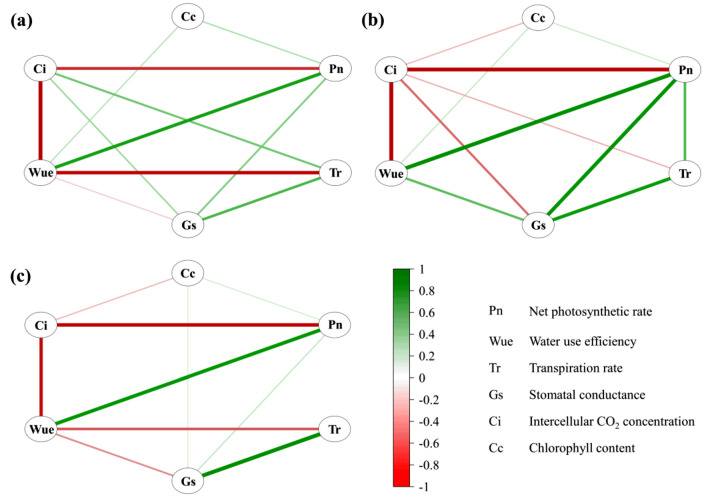
Correlation networks among photosynthetic traits in F_2_ population. (**a**–**c**) are the correlation networks for the seedling stage (n = 281), budding stage (n = 255) and filling stage of primary spike (n = 254), respectively. Only significant correlations (*p* < 0.05) are shown. The strength of the correlation is reflected by the thickness of the lines and is colored from green (coefficient = 1) to red (coefficient = − 1). For details, see Supplementary Table S1.

Pn was positively correlated with Wue, Gs and Cc and negatively with Ci at 3 stages, and positively with Tr at stage I (Fig. 2, Supplementary Table S1). 3 linear regression equations were built corresponding to 3 stages respectively, i.e.,

$$\begin{aligned} {\text{Pn }} & = \, - {13}.{732 } + { 4}.{\text{169Wue }} + { 2}.{\text{779Tr }} + { 7}.0{\text{35Gs}}, \\ {\text{Pn }} & = \, - {4}.0{93 } + { 3}.{\text{314Wue }} + { 2}.{\text{389Tr }} + { 2}0.{\text{725Gs }} - \, 0.0{\text{19Ci}}, \\ {\text{Pn }} & = \, - {16}.{735 } + { 3}.{\text{859Wue }} + { 4}.{\text{199Tr}}. \\ \end{aligned}$$

It could be seen that Wue played the most important positive role on Pn at 3 stages, followed by Tr.

The path analysis revealed that Wue and Tr were critical variables affecting Pn (Table 2), Wue was the important decision factor and Tr was the important limiting factor. The negative correlation between Wue and Tr at stages I and III but positive correlation at stage II resulted in the negative decision coefficient of Tr at stages I and III but positive at stage II, it seemed that the contradiction between Wue and Tr reached a relatively harmonious state at stage II.

**Table 2:** Path coefficients in F_2_ population.

Stage	Trait^a^	Correlation coefficient	Path coefficient^b^					Decision coefficient
			Wue	Tr	Gs	Ci	Total ^c^	
I	Wue	0.631	1.189	− 0.536	− 0.022	–	− 0.558	0.087
	Tr	0.051	− 0.807	0.790	0.068	–	− 0.738	− 0.543
	Gs	0.339	− 0.188	0.387	0.140	–	0.199	0.075
II	Wue	0.849	0.612	0.022	0.109	0.106	0.237	0.665
	Tr	0.544	0.045	0.308	0.161	0.031	0.236	0.240
	Gs	0.809	0.307	0.228	0.217	0.057	0.592	0.304
	Ci	− 0.828	− 0.528	− 0.077	− 0.101	− 0.123	− 0.705	0.188
III	Wue	0.755	1.102	− 0.347	–	–	− 0.347	0.450
	Tr	0.123	− 0.560	0.683	–	–	− 0.560	− 0.298

^a^The trait description is the same as in Table 1.

^b^Path coefficient: the underlined ones are direct path coefficients, and the rest is the indirect path coefficients of one independent variable on Pn through other independent variables; “-” indicates a missing value.

^c^Total: total indirect path coefficient.

### Genetic map construction

A total of 63/42 (F_2_/BC_1_, the same below) pairs of polymorphic SSR primers with clear and stable bands were selected from 1750 pairs of SSR primers. At the LOD threshold of 7.0/9.0, 59/31 polymorphic SSR primers were grouped into 9/4 linkage groups, encompassing 629/360 cM of the genome, with an average marker interval of 10.66/11.61 cM (Supplementary Fig. S1).

### QTL mapping

**Figure 3:**
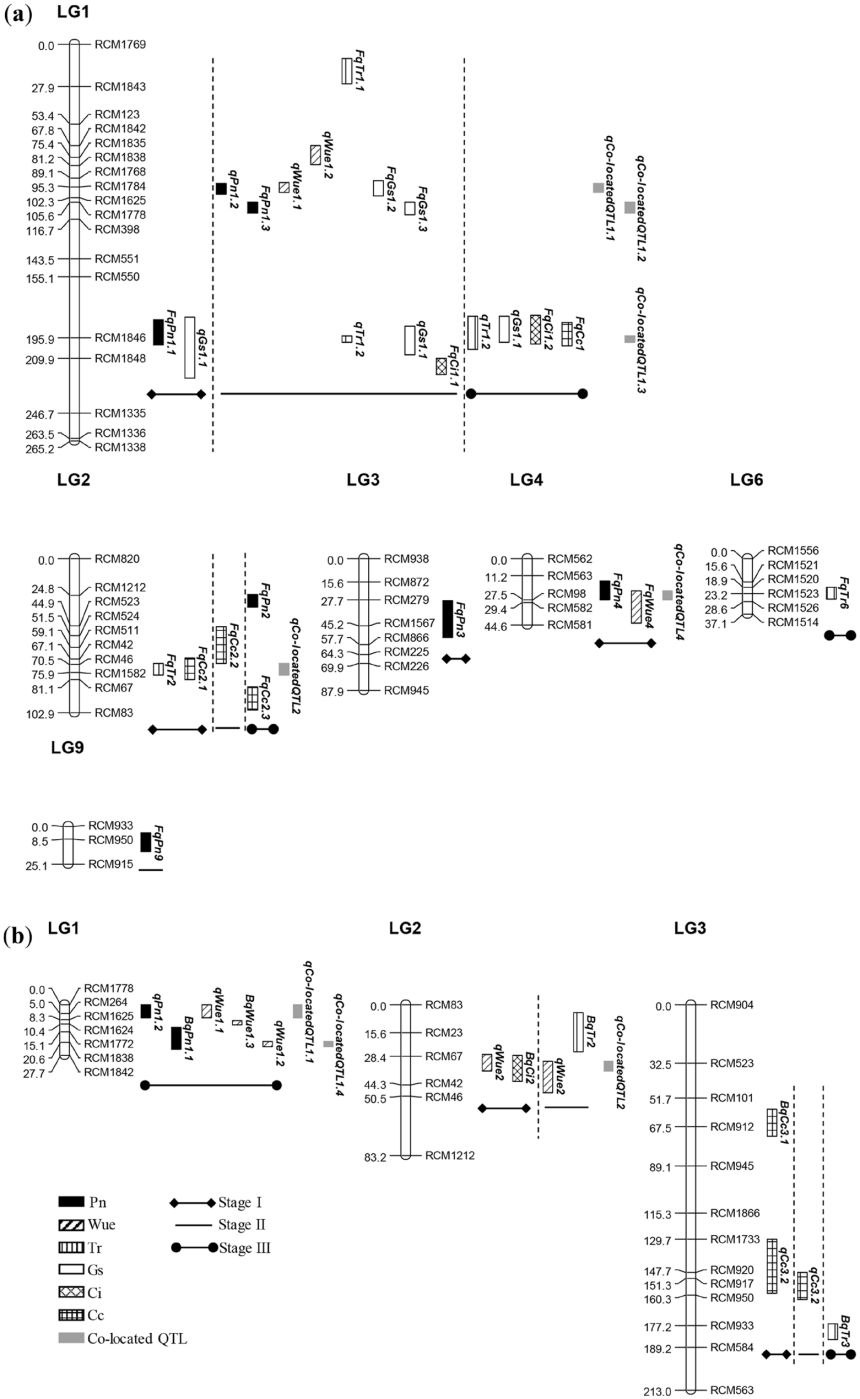
QTL distribution map in populations F_2_ (**a**) and BC_1_ (**b**). The trait description is the same as in Table 1.

A total of 26 QTLs were identified at 3 stages in F_2_ population (Fig. 3, Table 3), including 3/3/1 (stage I/II/III, the same below), 1/2/0, 1/2/2, 1/3/1, 0/1/1, and 1/1/2 QTLs conferring Pn, Wue, Tr, Gs, Ci and Cc, with a PVE of 8.41%/8.91%/6.17%, 5.36%/31.17%/0, 7.31%/12.80%/15.15%, 1.60%/6.44%/0.02%, 0/1.10%/0.70% and 2.77%/3.96%/6.50% respectively. The PVE of single QTL ranged from 0.10–7.10%, 5.36–16.71%, 0.19–12.61%, 0.02–4.91%, 0.70–1.10% and 1.23–5.27% respectively. Numerous minor QTLs were identified, only 4 QTLs, *qWue1.1*, *qWue1.2*, *FqTr1.1* and *FqTr6*, with a PVE over 10% and focused on the 2 traits, Wue and Tr*.*

**Table 3:** QTLs mapped in F_2_ population.

Stage	Trait	QTL	LG	Position (cM)	LOD	Additive	Dominant	PVE (%)	Confidence interval (cM)	Marker interval
I	Pn	*FqPn3*	3	40.71	2.32	− 0.85	1.79	4.88	28.4–53	RCM279–RCM866
		*FqPn1.1*	1	195.91	2.59	1.32	0.76	1.88	184–201.2	RCM550–RCM1848
		*FqPn4*	4	27.21	2.39	1.50	0.77	1.64	15.2–27.6	RCM563–RCM98
	Wue	*FqWue4*	4	38.41	2.94	0.60	0.07	5.36	21.4–43.4	RCM563–RCM581
	Tr	*FqTr2*	2	73.51	2.55	− 0.71	0.18	7.31	70.3–78.2	RCM46–RCM67
	Gs	*qGs1.1*	1	203.91	2.30	− 0.03	− 0.02	1.60	182.3–223	RCM550–RCM1335
	Cc	*FqCc2.1*	2	68.11	2.43	1.00	0.24	2.77	66.8–80.9	RCM42–RCM67
II	Pn	*FqPn9*	9	8.51	2.56	− 2.16	3.46	7.10	4.3–16.8	RCM933–RCM915
		*qPn1.2*	1	95.31	3.28	− 0.50	− 4.37	1.71	92.8–100.3	RCM1768–RCM1625
		*FqPn1.3*	1	108.61	4.45	− 6.18	− 4.49	0.10	105.5–112.7	RCM1778–RCM398
	Wue	*qWue1.1*	1	95.21	3.00	0.62	− 0.75	16.71	92.4–98.8	RCM1768–RCM1625
		*qWue1.2*	1	71.81	2.02	− 0.90	− 1.10	14.46	67.8–80.4	RCM1842–RCM1838
	Tr	*FqTr1.1*	1	17.01	4.84	− 0.87	− 0.32	12.61	9.4–26.1	RCM1769–RCM1843
		*qTr1.2*	1	195.91	3.52	0.06	0.61	0.19	195.1–199.4	RCM1846–RCM1848
	Gs	*FqGs1.2*	1	95.31	2.85	0.01	− 0.04	4.91	91.3–101.3	RCM1768–RCM1625
		*FqGs1.3*	1	108.61	3.85	− 0.05	− 0.05	1.39	105.5–114.2	RCM1778–RCM398
		*qGs1.1*	1	195.11	4.65	0.02	0.05	0.14	188.2–207.5	RCM550–RCM1848
	Ci	*FqCi1.1*	1	209.91	2.29	26.34	10.45	1.10	209.9–220.5	RCM1848–RCM1335
	Cc	*FqCc2.2*	2	65.11	2.09	1.20	− 0.09	3.96	45.6–70.3	RCM523–RCM46
III	Pn	*FqPn2*	2	24.81	2.78	2.42	− 0.38	6.17	24–32.3	RCM820–RCM523
	Tr	*FqTr6*	6	22.91	2.30	− 0.26	0.37	12.24	19.1–26.7	RCM1520–RCM1526
		*qTr1.2*	1	195.91	2.64	− 0.32	− 0.10	2.91	181.9–203.7	RCM550–RCM1848
	Gs	*qGs1.1*	1	195.91	3.40	− 0.01	− 0.04	0.02	181.6–199	RCM550–RCM1848
	Ci	*FqCi1.2*	1	194.11	4.02	14.29	24.09	0.70	180.9–200.5	RCM550–RCM1848
	Cc	*FqCc2.3*	2	99.21	2.75	0.78	− 1.74	5.27	85.6–101.2	RCM67–RCM83
		*FqCc1*	1	195.91	3.00	0.28	− 2.33	1.23	185.8–201.8	RCM550–RCM1848

A total of 13 QTLs were identified in BC_1_ population (Fig. 3, Table 4), just the half of F_2_ population, including 0/0/2, 1/1/3, 0/1/1, 0/0/0, 1/0/0 and 2/1/0 QTLs conferring Pn, Wue, Tr, Gs, Ci and Cc, with a PVE of 0/0/12.41%, 3.82%/6.40%/27.88%, 0/4.92%/10.97%, 0/0/0, 4.33%/0/0 and 20.14%/4.08%/0 respectively. The PVE of single QTL ranged from 5.55–6.86%, 3.82–12.58%, 4.92–10.97%, 0, 4.33% and 4.08–14.05% respectively. Although the identified QTLs were less, the PVE of each QTL was generally enlarged. In spite of this, only the PVE of *BqCc3.1*, *qWue1.1*, *BqWue1.3* and *BqTr3* exceeded or approached 10%. Except for *BqCc3.1*, the main-effect QTLs still focused on the 2 traits, Wue and Tr.

**Table 4:** QTLs mapped in BC_1_ population.

Stage	Trait	QTL	LG	Position (cM)	LOD	Additive	PVE (%)	Confidence interval (cM)	Marker interval
I	Wue	*qWue2*	2	28.41	2.12	0.85	3.82	27.6–36.5	RCM67–RCM42
	Ci	*BqCi2*	2	30.41	2.21	− 27.18	4.33	27.9–42.3	RCM67–RCM42
	Cc	*BqCc3.1*	3	64.81	4.73	4.03	14.05	58–72.7	RCM101–RCM945
		*qCc3.2*	3	140.71	2.63	− 2.07	6.09	129.6–159.5	RCM1733–RCM950
II	Wue	*qWue2*	2	40.41	2.50	1.46	6.40	31.3–48.7	RCM67–RCM46
	Tr	*BqTr2*	2	15.61	2.36	0.56	4.92	4.2–26	RCM83–RCM67
	Cc	*qCc3.2*	3	151.31	2.00	− 2.13	4.08	147.7–162.7	RCM920–RCM933
III	Pn	*qPn1.2*	1	2.01	2.45	− 7.32	6.86	0–7.3	RCM1778–RCM1625
		*BqPn1.1*	1	20.61	2.34	6.14	5.55	12.4–24.7	RCM1624–RCM1842
	Wue	*qWue1.1*	1	4.01	3.07	− 4.05	12.58	0–7	RCM1778–RCM1625
		*BqWue1.3*	1	10.31	3.45	− 4.52	9.28	8.8–10.9	RCM1625–RCM1624
		*qWue1.2*	1	20.61	2.52	3.65	6.02	20.1–23	RCM1838–RCM1842
	Tr	*BqTr3*	3	177.21	2.51	− 0.92	10.97	176.3–184.8	RCM950–RCM584

*qTr1.2* and *qGs1.1* were stable QTLs identified in F_2_ population, the former was simultaneously detected at stages II and III, the latter at 3 stages. *qWue2* and *qCc3.2* were stable QTLs in BC_1_ population, all detected at stages I and II. 3 stable QTLs, *qWue1.1*, *qWue1.2* and *qPn1.2*, were jointly identified in 2 populations, with a PVE of 16.71%/12.58% (F_2_/BC_1_, the same below), 14.46%/6.02% and 1.71%/6.86% respectively.

6 co-located QTLs were identified, each contained 2–8 alleles, shared by 2–5 traits (Table 5), which proved that the gene pleiotropy or close linkage between genes was common between the photosynthetic traits and provided genetic basis for genetically correlated selection in the breeding for high photosynthetic efficiency.

**Table 5:** Information of co-located QTLs.

Co-located QTL	*qCo-locatedQTL1.1*	*qCo-locatedQTL1.2*	*qCo-locatedQTL1.3*	*qCo-locatedQTL1.4*	*qCo-locatedQTL2*	*qCo-locatedQTL4*
Population	F_2_/BC_1_	F_2_	F_2_	F_2_/BC_1_	F_2_/BC_1_	F_2_
Overlap interval	92.8–98.8/0–7.0	105.5–112.7	195.1–199.0	67.8–80.4/20.1–23.0	70.3–78.2/31.3–36.5	21.4–27.6
Marker interval	RCM1768-RCM1625/RCM1778-RCM1625	RCM1778-RCM398	RCM1846-RCM1848	RCM1842-RCM1838/RCM1772-RCM1842	RCM46-RCM67/RCM67-RCM42	RCM563-RCM98
LOD	2.45–3.28/2.45–3.07	3.85–4.45	2.3–4.65	2.02/2.34–2.52	2.43–2.55/2.12–2.50	2.39–2.94
PVE (%)	1.71–16.71/6.86–12.58	0.10–1.39	0.02–2.91	14.46/5.55–6.02	2.77–7.31/3.82–6.40	1.64–5.36
Shared by	Pn, Wue, Gs	Pn, Gs	Pn, Tr, Gs, Ci, Cc	Wue/Pn, Wue	Tr, Cc/Wue, Ci	Pn, Wue
Allele	*qPn1.2*, *qWue1.1*, *FqGs1.2*/*qPn1.2*, *qWue1.1*	*FqPn1.3*, *FqGs1.3*	*FqPn1.1*, *qGs1.1*, *qTr1.2*, *FqCi1.2*, *FqCc1*	*qWue1.2*/*BqPn1.1*, *qWue1.2*	*FqTr2*, *FqCc2.1*/*qWue2*, *BqCi2*	*FqPn4*, *FqWue4*
Allele number	3/2	2	8	1/2	2/3	2
Stage	II/II, III	II	I, II, III	II/III	I/I, II	I

2 QTL clusters, located within the marker intervals RCM1842-RCM1335 and RCM523- RCM83, contained 15/5 and 4/4 QTLs conferring multiple traits, including co-located QTLs and main-effect QTLs (Table 6).

**Table 6:** Information of 2 QTL clusters.

QTL cluster	QTL cluster 1 (RCM1842-RCM1335)	QTL cluster 2 (RCM523-RCM83)
Population	F_2_/BC_1_	F_2_/BC_1_
LOD	2.02–4.65/2.34–3.45	2.09–2.75/2.12–2.50
PVE (%)	0.02–16.71/5.55–12.58	2.77–7.31/3.82–6.40
Shared by	Pn, Wue, Tr, Gs, Ci, Cc/Pn, Wue	Tr, Cc/Wue, Tr, Ci
QTL	*qCo-locatedQTL1.1*, *qCo-locatedQTL1.2*, *qCo-locatedQTL1.3*, *FqCi1.1*/*qCo-locatedQTL1.1*, *qCo-locatedQTL1.4*, *BqWue1.3*	*qCo-locatedQTL2*, *FqCc2.2*, *FqCc2.3*/*qCo-locatedQTL2*, *BqTr2*
QTL number^a^	15/5	4/4
PVE > 5% QTL number	2/5	2/1
Main-effect QTL	*qWue1.1*, *qWue1.2*/*qWue1.1*	–

^a^A co-located QTL contains more than one QTLs.

### Analysis of epistatic QTLs

To estimate the genetic share of epistatic effect, the QTLNetwork 2.0 was utilized to identify the single-locus QTL (Supplementary Table S2) and the epistatic QTLs. The single-locus QTLs were much less than those identified with WinQTLCart v2.5, but the QTLs except *FqCc3.1*, *FqCc5.1*, *BqCc1.1* and *BqCc3.3* were all detected too.

A total of 53 (31 pairs) epistatic QTLs were detected at 3 stages in F_2_ population (Table 7, Supplementary Fig. S1), including 2/2/6 (stage I/II/III, the same below), 5/6/3, 4/4/2, 6/3/2, 3/2/0 and 3/0/0 ones conferring Pn, Wue, Tr, Gs, Ci and Cc, with a PVE of 6.52%/6.47%/19.04%, 16.72%/15.67%/14.12%, 18.57%/15.58%/7.34%, 21.72%/8.52%/7.13%, 13.33%/4.94%/0 and 7.84%/0/0 respectively, and the PVE of each pairwise QTLs ranged from 1.56–6.52%, 1.93–8.11%, 4.42–9.08%, 0.88–8.20%, 4.94–7.14% and 4.37–3.47% respectively. Except for epistatic effects, 3 QTLs, i.e., *qPn1.2*, *FqGs1.2* and *FqCi1.2*, had single locus effects also.

**Table 7:** Epistasis QTLs identified in F_2_ population.

Stage	Trait	QTL_i	Interval_i	Position_i (cM)	Range_i (cM)	QTL_j	Interval_j	Position_j (cM)	Range_j (cM)	PVE (%)	AA	AD	DA	DD
I	Pn	*FqPn2.2*	RCM820–RCM1212	9	0.0–14.0	*FqPn7*	RCM150–RCM1824	9	3.0–15.0	6.52	− 0.02	− 2.13	− 0.78	− 8.56
	Wue	*FqWue1.4*	RCM1842–RCM1835	67.8	63.4–69.8	*FqWue1.7*	RCM550–RCM1846	187.1	177.1–196.9	7.56	− 0.12	1.88	0.53	− 2.20
		*FqWue1.5*	RCM1838–RCM1768	88.2	84.2–88.2	*FqWue1.7*	RCM550–RCM1846	187.1	177.1–196.9	3.77	− 0.09	− 1.09	1.47	− 0.17
		*FqWue1.6*	RCM1848–RCM1335	214.9	206.9–225.9	*FqWue6*	RCM1521–RCM1520	17.6	10.0–23.2	5.39	− 0.68	− 0.49	1.67	− 0.24
	Tr	*FqTr2.2*	RCM523–RCM524	44.9	39.8–46.9	*FqTr9*	RCM933–RCM950	4	0.0–8.0	8.43	− 0.60	0.17	0.03	0.93
		*FqTr2.3*	RCM511–RCM42	60.1	56.5–65.1	*FqTr9*	RCM933–RCM950	4	0.0–8.0	4.42	0.05	0.71	0.59	0.83
		*FqTr4*	RCM563–RCM98	14.2	5.0–22.2	*FqTr9*	RCM933–RCM950	4	0.0–8.0	5.72	0.93	− 0.44	− 0.46	0.00
	Gs	*FqGs1.4*	RCM1843–RCM123	31.9	25.0–37.9	*FqGs1.6*	RCM1842–RCM1835	67.8	67.4–72.8	7.49	0.04	0.00	− 0.03	0.15
		*FqGs1.5*	RCM1848–RCM1335	244.9	236.9–252.7	*FqGs2.1*	RCM820–RCM1212	3	0.0–11.0	8.20	− 0.06	− 0.06	0.04	− 0.01
		*FqGs5*	RCM76–RCM74	29.6	18.9–30.6	*FqGs9*	RCM950–RCM915	20.5	10.5–24.5	6.03	− 0.01	0.08	− 0.03	− 0.02
	Ci	*FqCi1.3*	RCM1842–RCM1835	69.8	67.4–71.8	***FqCi1.2***	RCM550–RCM1846	193.1	183.1–199.9	6.19	− 1.27	− 45.82	1.33	− 4.27
		***FqCi1.2***	RCM550–RCM1846	193.1	183.1–199.9	*FqCi2.1*	RCM820–RCM1212	16	9.0–24.8	7.14	− 6.06	− 39.20	25.78	56.18
	Cc	*FqCc1.2*	RCM1769–RCM1843	6	0.0–14.0	*FqCc1.4*	RCM398–RCM551	122.7	112.6–131.7	4.37	0.87	− 3.80	− 0.39	− 4.00
		*FqCc1.3*	RCM1769–RCM1843	24	16.0–44.9	*FqCc1.4*	RCM398–RCM551	122.7	112.6–131.7	3.47	0.71	− 0.90	2.02	2.07
II	Pn	***qPn1.2***	RCM1784–RCM1625	95.3	92.1–98.3	*FqPn9*	RCM950–RCM915	12.5	5.0–18.5	6.47	4.05	1.34	1.87	5.54
	Wue	*FqWue1.8*	RCM1843–RCM123	47.9	37.9–56.4	*FqWue3.1*	RCM1567–RCM866	45.2	37.7–50.2	5.63	0.24	− 0.59	− 0.71	0.56
		*qWue1.2*	RCM1842–RCM1835	74.8	72.8–74.8	*FqWue3.2*	RCM279–RCM1567	43.7	37.7–50.2	1.93	0.16	− 0.97	0.74	− 0.09
		*FqWue1.9*	RCM398–RCM551	128.7	118.7–135.7	*FqWue9*	RCM950–RCM915	17.5	12.5–23.5	8.11	0.56	1.33	0.78	1.38
	Tr	*FqTr1.3*	RCM1336–RCM1338	264.5	254.7–264.5	*FqTr2.2*	RCM523–RCM524	50.9	47.9–52.5	6.50	− 0.47	− 0.43	− 0.20	1.17
		*FqTr2.4*	RCM67–RCM83	91.1	83.1–101.1	*FqTr3.1*	RCM872–RCM279	25.6	20.6–33.7	9.08	− 0.43	0.69	− 1.18	0.36
	Gs	*FqGs1.6*	RCM1842–RCM1835	72.8	71.8–74.8	*FqGs9*	RCM950–RCM915	8.5	2.0–15.5	7.64	0.09	− 0.02	− 0.03	0.09
		***FqGs1.2***	RCM1784–RCM1625	95.3	92.1–99.3	*FqGs9*	RCM950–RCM915	8.5	2.0–15.5	0.88	0.01	0.00	0.03	− 0.01
	Ci	*FqCi1.4*	RCM1784–RCM1625	95.3	91.1–98.3	*FqCi2.2*	RCM524–RCM511	51.5	50.9–54.5	4.94	10.90	− 22.34	23.25	55.52
III	Pn	*FqPn1.4*	RCM1843–RCM123	41.9	34.9–48.9	*FqPn2.3*	RCM1212–RCM523	39.8	33.8–46.9	5.77	− 0.80	− 0.54	− 2.76	6.94
		*FqPn1.5*	RCM1842–RCM1835	67.8	65.4–68.8	*FqPn2.3*	RCM1212–RCM523	39.8	33.8–46.9	5.19	1.41	2.91	− 4.47	− 1.33
		*FqPn2.3*	RCM1212–RCM523	39.8	33.8–46.9	*FqPn4*	RCM98–RCM582	27.5	21.2–29.4	6.52	− 3.37	− 0.50	− 1.24	− 3.88
		*FqPn2.4*	RCM511–RCM42	66.1	61.1–66.1	*FqPn4*	RCM98–RCM582	27.5	21.2–29.4	1.56	− 0.74	− 1.49	0.27	− 2.83
	Wue	*FqWue1.8*	RCM1843–RCM123	44.9	37.9–55.4	*FqWue2*	RCM1212–RCM523	24.8	16.0–31.8	7.97	− 0.36	0.56	− 0.93	1.22
		*FqWue1.4*	RCM1842–RCM1835	67.8	64.4–69.8	*FqWue2*	RCM1212–RCM523	40.8	34.8–47.9	6.15	0.90	0.83	− 0.90	− 0.40
	Tr	*FqTr1.4*	RCM1769–RCM1843	0	0.0–6.0	*FqTr3.2*	RCM226–RCM945	76.9	69.9–82.9	7.34	− 0.25	0.29	0.43	− 1.16
	Gs	*FqGs1.7*	RCM123–RCM1842	60.4	58.4–63.4	*FqGs2.2*	RCM42–RCM46	69.1	63.1–75.5	7.13	0.04	0.10	0.04	− 0.04

The underlined loci have both epistatic and single-locus effects.

A total of 15 (8 pairs) epistatic QTLs in BC_1_ population were identified (Table 8, Supplementary Fig. S1), including 4/0/2, 0/2/0, 0/0/2, and 0/3/2 ones conferring Pn, Gs, Ci and Cc, with a PVE of 8.02%/0/5.70%, 0/5.10%/0, 0/0/5.97% and 0/3.52%/2.79% respectively, and the PVE of each pairwise QTLs ranged from 3.57–5.70%, 5.10%, 5.97% and 0.26–3.26% respectively. No epistatic QTL underlying Wue and Tr was detected. Among all the epistatic QTLs, *qCc3.2* was the only one with single locus effect. In most cases, the epistatic effect was the major genetic component of the photosynthetic traits (Table 9).

**Table 8:** Epistasis QTLs identified in BC_1_ population.

Stage	Trait	QTL_i	Interval_i	Position_i (cM)	Range_i (cM)	QTL_j	Interval_j	Position_j (cM)	Range_j (cM)	PVE (%)	AA
I	Pn	*BqPn2.1*	RCM67–RCM42	28.4	27.6–39.4	*BqPn3.1*	RCM950–RCM933	160.3	152.3–168.3	3.57	− 3.38
		*BqPn2.2*	RCM46–RCM1212	82.5	57.5–82.5	*BqPn3.2*	RCM933–RCM584	189.2	181.2–192.2	4.45	7.21
II	Gs	*BqGs1*	RCM1624–RCM1772	10.4	8.3–13.4	*BqGs3*	RCM933–RCM584	182.2	175.3–189.2	5.10	0.09
	Cc	*BqCc1.1*	RCM1778–RCM264	0	0.0–20.6	***qCc3.2***	RCM917–RCM950	153.3	147.7–169.3	3.26	0.98
		*BqCc1.2*	RCM1624–RCM1772	10.4	0.0–20.6	***qCc3.2***	RCM917–RCM950	153.3	147.7–169.3	0.26	1.20
III	Pn	*BqPn3.3*	RCM917–RCM950	151.3	140.7–169.3	*BqPn4*	RCM522–RCM74	9.4	6.3–17.4	5.70	− 4.97
	Ci	*BqCi3*	RCM920–RCM917	149.7	140.7–160.3	*BqCi4*	RCM522–RCM74	9.4	6.3–17.4	5.97	47.37
	Cc	*BqCc1.1*	RCM1778–RCM264	0	0.0–27.6	*BqCc3.3*	RCM950–RCM933	160.3	147.7–174.3	2.79	2.58

*qCc3.2* has both epistatic and single-locus effects.

**Table 9:** Percentage of epistatic effect.

Stage	Trait	F_2_ population			BC_1_ population		
		SE (%)	EE (%)	P (%)	SE (%)	EE (%)	P (%)
I	Pn	0	6.52	100.00	0	8.02	100.00
	Wue	5.04	16.72	76.84	0	0	–
	Tr	0	18.57	100.00	0	0	–
	Gs	0	21.72	100.00	0	0	–
	Ci	0	13.33	100.00	0	0	–
	Cc	3.53	7.84	68.95	10.18	0	0
II	Pn	5.10	6.47	55.92	0	0	–
	Wue	5.03	15.67	75.70	0	0	–
	Tr	11.70	15.58	57.11	0	0	–
	Gs	13.08	8.52	39.44	0	5.10	100.00
	Ci	0	4.94	100.00	0	0	–
	Cc	6.21	0	0	0	3.52	100.00
III	Pn	0	19.04	100.00	0	5.70	100.00
	Wue	0	14.12	100.00	0	0	–
	Tr	0	7.34	100.00	0	0	–
	Gs	6.64	7.13	51.78	0	0	–
	Ci	5.30	0	0	0	5.97	100.00
	Cc	9.59	0	0	2.38	2.79	53.97

*SE* PVE of single locus effect, *EE* PVE of epistasis effect, *P* percentage of epistatic effect.

### Gene annotation

16 and 30 ORFs (Open reading frame) were retrieved within the confidence interval of *qWue1.2* and *FqTr6*, 13 and 22 of which were successfully annotated (Supplementary Table S3). Combining the genome retrieval results and literature description, 2 possible candidate genes (*29864.m001449*, *29864.m001459*) underlying *qWue1.2* were annotated as GDSL esterase/lipase (GDSL) and polyamine oxidase 1 (PAO1), the former catalyzed the polymerization of the leaf cuticle which covered the surface of aerial organs and assisted in avoiding water loss^37, 38^, the latter resulted in the production of H_2_O_2_, which closed the stomata on the blades^39, 40^. 3 possible candidate genes (*29822.m003500, 29822.m003505*, *29822.m003509*) underlying *FqTr6* were annotated as homeobox-leucine zipper protein ATHB-20 (ATHB-20)*,* calcineurin B-like protein 9 (CBL9) and casein kinase II subunit beta-1 (CKB1), functioning in the formation of vascular network^41^, regulating stomatal behaviour^42, 43^ and stomatal aperture^44^ in *Arabidopsis* respectively.

## Discussions

The 6 photosynthetic traits were all quantitative traits controlled by major genes and polygene together (Fig. 1). Limited to present conditions, it is difficult to trace the minor genes, while major genes, with obvious selection effect and easy to be genetically operated, are favored by breeders in breeding practice. In this study, 3–9 QTLs were mapped for each photosynthetic trait, most of them with a minor contribution rate. Fortunately, a few main-effect QTLs were found, which are expected to play important roles in improving selection efficiency in breeding. Epistasis effect, accounting for much larger proportion of the PVE than additive and dominant effects, was the major genetic component of photosynthetic traits (Table 8).

No main-effect QTL underlying Pn was identified, it meant that it is difficult to achieve ideal selection effect for Pn by molecular marker-assisted selection. However, 6 main-effect QTLs, i.e., *qWue1.1*, *qWue1.2*, *FqTr1.1*, *FqTr6, BqTr3* and *BqCc3.1* were found, the former 5 focused on 2 traits, Wue and Tr, and among the 6 co-located QTLs, 3 were shared by Pn and Wue, 1 was shared by Pn and Tr. Since Wue and Tr were the important decision factor and limiting factor affecting Pn respectively, and regulating Wue and Tr could increase photosynthetic capacity^8, 9, 20^, these main-effect QTLs could be used as a method of genetically correlated selection for Pn.

Most of the QTLs conferring the photosynthetic traits varied at different stages, demonstrating that the genes controlling photosynthesis are not all the same at different stages and in different environments, which increased the difficulty of the breeding for high photosynthetic efficiency. As a vital activity of plant life, photosynthesis must be controlled by an extremely complex genetic system, including constitutively expressed house-keeping genes and inducible luxury genes. In all the identified QTLs, 7 were stable QTLs (Tables 3, 4). Among them, some such as *qGs1.1* was detected at all the 3 stages, some such as *qWue2*, *qTr1.2* and *qCc3.2* at 2 stages, and some such as *qWue1.1*, *qWue1.2* and *qPn1.2* in 2 populations, this not only explained the reliability of the mapping results to a certain extent, but also provided some clues for the discovery of house-keeping genes.

6 co-located QTLs were identified in this study, the allelic QTLs of each were located within an interval of 3–7 cM, with a similar PVE (Fig. 3, Table 5), which was similar to the results reported in maize by Xie et al.^22^. The existence of co-located QTLs indicated that the pleiotropy of genes (at least close linkage between genes) was common between photosynthetic traits and the genetic foundation of significant correlationship between photosynthetic traits (Fig. 2). Sometimes, a main-effect QTL was likely to be both stable QTL and co-located QTL. For example, *qWue1.1* and *qWue1.2*, detected in F_2_ population and BC_1_ population simultaneously, with a PVE of 16.71%/12.58% and 14.46%/6.02% respectively, were the allelic member of *qCo-locatedQTL1.1* and *qCo-locatedQTL1.4* respectively.

In summary, the above results will enhance our understanding of the genetic structure for photosynthetic traits in castor and lay the foundation for genetically correlated selection. Breeding superior cultivars or accessions with high photosynthetic efficiency will benefit greatly from analyzing whether the 5 predicted candidate genes affect stomatal behaviour in blades or photosynthetic carbon gain through transgenic experiments, and exploring their mechanism of functions.

## Materials and methods

### Materials

The populations F_1_, F_2_ and BC_1_ derived from the cross of 9048 (P_1_) × 16-201 (P_2_) were used in this study. 9048 was a pistillate line with tall and compact plant architecture, stout stalks, large spikes, medium-sized seeds and a medium seed setting rate, it was the female parent of Zibi 5, a main cultivar in China. 16-201 was a monecious line with a dwarf plant type, thin and tough stalks, multiple scattered branches, multiple effective spikes and high seed setting rate, but small spike, capsule and seed size. The population size of P_1_, P_2_, F_1_, F_2_ and BC_1_ (F_1_ × P_2_) was 25, 25, 25, 282 and 250 respectively. They were planted at the experimental base of Guangdong Ocean University in Zhanjiang, Guangdong, China in Sep. 2020, with a plant row spacing of 1 m. The cultivation management was the same as high yield field.

### Phenotype investigation

The net photosynthetic rate (Pn), transpiration rate (Tr), stomatal conductance (Gs) and intercellular CO_2_ concentration (Ci) were measured at 3 stages, i.e., the seedling stage (the fully unfolded 5th leaf, stage I), the budding stage (the fully unfolded leaf below the primary spike, stage II) and the filling stage of primary spike (the fully unfolded leaf below the 1st primary branching spike, stage III), on a sunny day between 8:30–11:30 am and 3:00–5:00 pm, using Li-6400 Portable Photosynthesis System (LI-COR, Lincoln, NB, USA) under photosynthetically active radiation of 1000 μmol m^−2^ s^−1^, air temperature of 25 °C, relative humidity of 60% and ambient CO_2_ concentration of 420 µmol CO_2_ mol^−1^. Water use efficiency (Wue) value was gained as the ratio of Pn and Tr according to specification. The measurements on each blade were repeated 5 times, with the mean of the 3 duplicate values after removing the maximum and minimum as the phenotypic value of each individual. The chlorophyll content (Cc) was measured using SPAD-502 Plus Chlorophyll Meter (Konica Minolta, Inc., Made in Japan), each blade was investigated 8–10 times, the average of which was used as the Cc value of the individual. Path analysis^45^ and Student’s *t* test were run with Software SPSS 25 and Excel 2010. The correlation network among photosynthetic traits was constructed using “qgraph” package in R^46^.

### DNA extraction and genotyping

The genomic DNA of each individual was extracted using the modified CTAB method^47^. The concentration and quality of DNA were examined using Ultra-micro UV–Vis Spectrophotometer (Micro drop, Made in China). DNA integrity was examined using electrophoresis on a 1.0% agarose gel. The genotyping of F_2_ and BC_1_ individuals was conducted using the polymerase chain reaction (PCR) technique with SSR (simple sequence repeats) primers. The PCR reaction system contained 0.5 μL each of SSR primers, 4.0 μL of 2 × PCR Mix, 1.0 μL of 30 ng μL^−1^ DNA, and 4.0 μL of ddH_2_O. PCR reactions and display of PCR products were performed following the procedure described by Yeboah et al.^48^.

### Genetic map construction

1750 pairs of SSR primers^49^ were used in genetic map construction. The polymorphic primers were screened out through the preliminary screening by parents DNA and the verification of small population and finally used to scan the whole population. The genetic map was constructed using the genotyping data of all the polymorphic primers by software QTLIcimapping v4.2 with Kosambi function.

### QTL mapping

QTLs were mapped using software WinQTLCart v2.5 with CIM (Composite interval mapping) method and a LOD threshold of 2.0. In order to detect the epistasis effects of QTLs, the software QTLNetwork v2.0 was used with the default parameters. Confidence intervals for all QTLs were determined at 95% confidence degree. The adjacent QTLs with overlapped confidence intervals and positions within 5 cM were regarded as the same QTL^50, 51^. The QTLs underlying the same trait detected simultaneously in different populations or at different stages were defined as the stable QTL. QTLs shared by more than 1 traits were defined as the co-located QTL. The QTLs with a PVE over 10% were considered as main-effect QTL. QTLs were named according to the rule of “q + trait abbreviation + linkage group serial number + QTL serial number on the linkage group”. In order to distinguish the QTLs identified in different populations, the capitals F and B were prefixed the QTLs detected in populations F_2_ and BC_1_ respectively, no any prefix before stable QTLs^51^. The co-located QTLs were additionally named as “q + Co-locatedQTL + linkage group serial number + QTL serial number”.

### Candidate gene prediction

Because the SSR primers used in this study were developed from the nucleotide sequence of the scaffolds^49^, with which the published castor genome framework was assembled^52^ (http://castorbean.jcvi.org/downloads.php), the possible candidate genes, covered by the main-effect QTLs with small physical distance from the linked SSR markers, could be retrieved with software IGB v9.1.8. BlastP functional annotation of all retrieved candidate genes was performed by Kobas 3.0 online tool (http://bioinfo.org/kobas). The trustworthy candidate genes were expected to be found through the combination of genomic annotation information and available literature description on them.

### Permission statement

9048 and 16-201 were stored at the castor research group of Guangdong Ocean University and used as the parental material in this study. The relevant report of wild material 16-201 has been registered in Guangdong Science and Technology Report Service with project number 2013B060400024 and report number 45625261X—2013B060400024/01, and 16-201 is jointly identified by the member of Guangdong Ocean University, Prof. Xuegui Yin, Dr. Jiannong Lu and Prof. Yuzhen Shi. All the experiments on plant resources, including the collection of castor germplasms, were performed following relevant local guidelines and regulations.

### Supplementary Information

Supplementary Information.

## Data Availability

The reference genomic databases are available in the published castor genome framework (http://castorbean.jcvi.org/downloads.php). The data that support the findings of this study are available on request from the corresponding author, Jiannong Lu, upon reasonable request.
