# Supplementary material for: Dynamic QTL mapping revealed primarily the genetic structure of photosynthetic traits in castor (Ricinus communis L.)
Source: Sci Rep. 2023 Aug 28;13:14071. doi: 10.1038/s41598-023-41241-y (PMC10462610; doi:10.1038/s41598-023-41241-y)
Supplement: Supplementary file 1 — Supplementary Information. [file 41598_2023_41241_MOESM1_ESM.pdf]

Dynamic QTL Mapping Revealed Primarily the Genetic Structure of Photosynthetic Traits in Castor (*Ricinus communis* L.)

Guanrong Huang, Xuegui Yin, Jiannong Lu*, Liuqin Zhang, Dantong Lin, Yu Xie, Haiyan Liu, Chaoyu Liu, Jinying Zuo, Xiaoxiao Zhang

College of Coastal Agricultural Sciences, Guangdong Ocean University, Zhanjiang, China, 524088

* Corresponding author: Jiannong Lu, lujnong@163.com

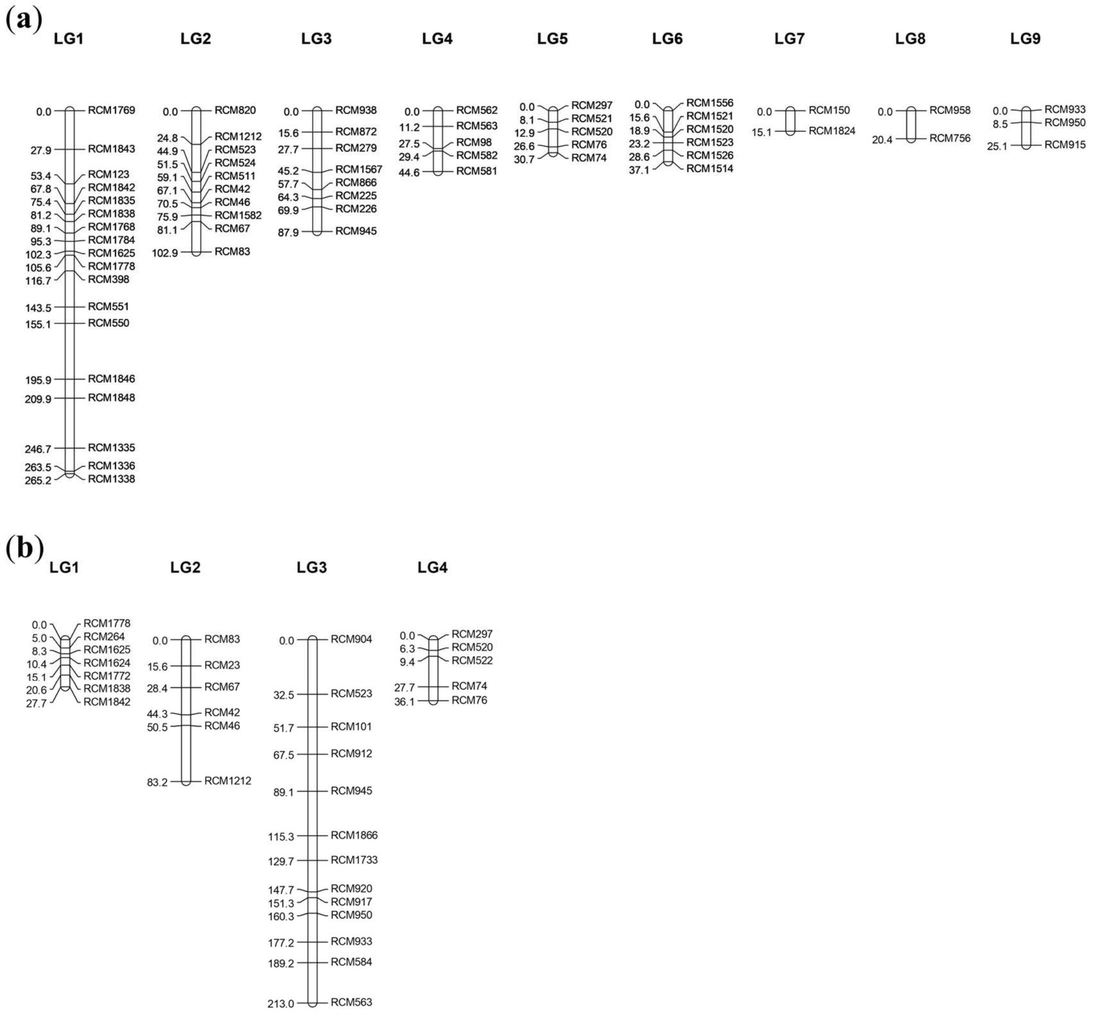

Supplementary Figure S1. The genetic linkage map of populations F_2 (a) and BC_1 (b)

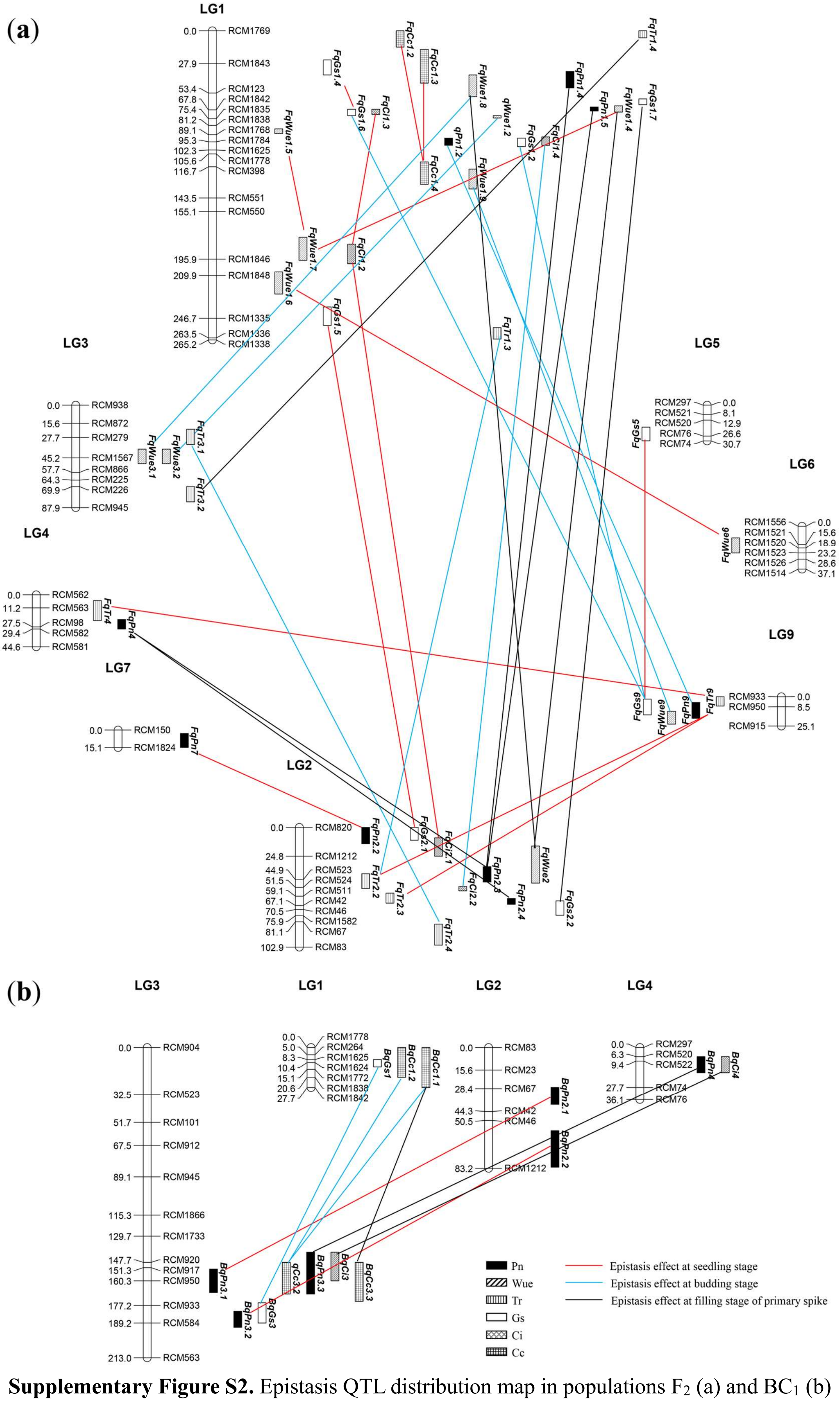

Supplementary Figure S2. Epistasis QTL distribution map in populations F_2 (a) and BC_1 (b)

The trait description is the same as in Table 1.

Supplementary Table S1. Correlation analysis between photosynthetic traits in F_2 population

Stage	Trait [a]	Pn	Wue	Tr	Gs	Ci
I	Wue	0.631**				
	Tr	0.051	-0.679**			
	Gs	0.339**	-0.158**	0.490**		
	Ci	-0.565**	-0.766**	0.386**	0.269**	
	Cc	0.228**	0.202**	-0.07	0.072	-0.114
II	Wue	0.849**				
	Tr	0.544**	0.073			
	Gs	0.809**	0.501**	0.742**		
	Ci	-0.828**	-0.862**	-0.250**	-0.464**	
	Cc	0.186**	0.196**	0.032	0.052	-0.232**
III	Wue	0.755**				
	Tr	0.123	-0.508**			
	Gs	0.197**	-0.343**	0.825**		
	Ci	-0.735**	-0.707**	0.09	-0.059	
	Cc	0.155*	0.122	0.026	0.138*	-0.216**

[a] The trait description is the same as in Table 1.

* refers to significant correlation, ** refers to extremely significant correlation.

Supplementary Table S2. QTLs mapped by QTLNetwork

Population	Stage	Trait	QTL	LG	Position (cM)	Additive	Dominant	PVE (%)	Confidence Interval (cM)	Marker Interval
F_2	I	Wue	*FqWue4*	4	39.4	0.60	0.10	5.04	27.5 - 44.4	RCM582 - RCM581
		Cc	*FqCc2.1*	2	68.1	1.02	0.44	3.53	61.1 - 75.5	RCM42 - RCM46
	II	Pn	*qPn1.2*	1	95.3	0.57	-4.33	5.1	92.1 - 98.3	RCM1784 - RCM1625
		Wue	*qWue1.1*	1	95.3	-0.21	-0.78	5.03	92.1 - 98.3	RCM1784 - RCM1625
		Tr	*FqTr1.1*	1	13	-0.58	-0.36	7.47	9.0 - 17.0	RCM1769 - RCM1843
			qTr1.2	1	195.9	0.04	0.50	4.23	181.1 - 198.9	RCM1846 - RCM1848
		Gs	*FqGs1.2*	1	95.3	0.01	-0.05	4.99	92.1 - 99.3	RCM1784 - RCM1625
			qGs1.1	1	195.9	0.02	0.05	8.09	188.1 - 197.9	RCM1846 - RCM1848
		Cc	*FqCc5.1*	5	30.6	0.99	-1.40	6.21	26.6 - 30.6	RCM76 - RCM74
	III	Gs	*qGs1.1*	1	195.9	-0.02	-0.03	6.64	184.1 - 197.9	RCM1846 - RCM1848
		Ci	*FqCi1.2*	1	192.1	9.46	26.23	5.3	180.1 - 200.9	RCM550 - RCM1846
		Cc	*FqCc1*	1	195.9	0.47	-2.71	6.19	189.1 - 199.9	RCM1846 - RCM1848
			FqCc3.1	3	86.9	-1.27	0.44	3.4	81.9 - 86.9	RCM226 - RCM945
BC_1	I	Cc	*BqCc3.1*	3	67.5	2.93	-	5.24	62.7 - 71.5	RCM912 - RCM945
			qCc3.2	3	135.7	-2.53	-	4.94	123.3 - 161.3	RCM1733 - RCM920
	III	Cc	*BqCc1.1*	1	0	-0.79	-	4.02	0.0 - 27.6	RCM1778 - RCM264
			BqCc3.3	3	119.3	-2.41	-	2.38	107.1 - 143.7	RCM1866 - RCM1733

Supplementary Table S3. Gene annotation

QTL	Candidate Gene	Gene symbol	Gene description
	29864.m001449	LOC8259276	GDSL esterase/lipase
	29864.m001450	LOC8259277	receptor-like protein EIX2
	29864.m001451	LOC107260732	phenylacetaldehyde reductase-like
	29864.m001452	LOC8259279	phenylacetaldehyde reductase
	29864.m001453	LOC8259280	phenylacetaldehyde reductase
	29864.m001454	LOC8259281	phenylacetaldehyde reductase
	29864.m001455	LOC8259282	pentatricopeptide repeat-containing protein At3g53170
qWue1.2	29864.m001456	LOC8285489	uncharacterized LOC8285489
	29864.m001457	LOC8259726	rhodanese-like domain-containing protein 4A, chloroplastic
	29864.m001459	LOC8259286	polyamine oxidase 1
	29864.m001461	LOC8259288	alpha-N-acetylglucosaminidase
	29864.m001462	LOC8259288	alpha-N-acetylglucosaminidase
	29864.m001463	LOC8259290	notchless protein homolog
	29864..m001464	LOC8259291	phenylacetaldehyde reductase
	29822.m003490	LOC8276042	galactose mutarotase
	29822.m003491	LOC8276043	3-dehydroquinate synthase homolog
	29822.m003492	LOC8276044	thyroid adenoma-associated protein homolog
	29822.m003493	LOC8276045	40S ribosomal protein S9-2
	29822.m003495	LOC8276047	tubulin alpha chain
	29822.m003496	LOC8276048	ABC transporter B family member 19
	29822.m003497	LOC8276049	transcription factor PRE1
	29822.m003498	LOC8276050	binding partner of ACD11 1
	29822.m003499	LOC8271329	uncharacterized protein At3g28850
	29822.m003500	LOC8276052	homeobox-leucine zipper protein ATHB-20
	29822.m003502	LOC8276054	60S ribosomal protein L30
	29822.m003503	LOC8276055	methanol O-anthraniloyltransferase
	29822.m003504	LOC8276056	serine/threonine-protein kinase SAPK3
FqTr6	29822.m003505	LOC8276057	calcineurin B-like protein 9
	29822.m003507	LOC8276058	coiled-coil domain-containing protein 97
	29822.m003508	LOC8276059	uncharacterized LOC8276059
	29822.m003509	LOC8276060	casein kinase II subunit beta-1
	29822.m003510	LOC8276061	uncharacterized LOC8276061
	29822.m003511	LOC8276062	60S ribosomal protein L37a
	29822.m003512	LOC8276063	SNF2 domain-containing protein CLASSY 3
	29822.m003513	LOC8276064	novel plant SNARE 11
	29822.m003514	LOC8276065	uncharacterized LOC8276065
	29822.m003515	LOC8276066	probable serine/threonine-protein kinase PBL19
	29822.m003516	LOC8276067	uncharacterized LOC8276067
	29822.m003517	LOC8276068	beta-amylase 3, chloroplastic
	29822.m003518	LOC8271322	uridylate-specific endoribonuclease B
	29822.m003519	LOC8271323	uncharacterized LOC8271323
